# Distinguishing mirror from glass: A “big data” approach to material perception

**DOI:** 10.1167/jov.22.4.4

**Published:** 2022-03-10

**Authors:** Hideki Tamura, Konrad Eugen Prokott, Roland W. Fleming

**Affiliations:** 1 Department of Computer Science and Engineering, Toyohashi University of Technology, Toyohashi, Aichi, Japan; 2 Department of Experimental Psychology, Justus Liebig University Giessen, Giessen, Germany; 3 Center for Mind, Brain and Behavior (CMBB), University of Marburg and Justus Liebig University Giessen, Germany

**Keywords:** material perception, neural networks, transparent material, gloss perception

## Abstract

Distinguishing mirror from glass is a challenging visual inference, because both materials derive their appearance from their surroundings, yet we rarely experience difficulties in telling them apart. Very few studies have investigated how the visual system distinguishes reflections from refractions and to date, there is no image-computable model that emulates human judgments. Here we sought to develop a deep neural network that reproduces the patterns of visual judgments human observers make. To do this, we trained thousands of convolutional neural networks on more than 750,000 simulated mirror and glass objects, and compared their performance with human judgments, as well as alternative classifiers based on “hand-engineered” image features. For randomly chosen images, all classifiers and humans performed with high accuracy, and therefore correlated highly with one another. However, to assess how similar models are to humans, it is not sufficient to compare accuracy or correlation on random images. A good model should also predict the characteristic errors that humans make. We, therefore, painstakingly assembled a diagnostic image set for which humans make systematic errors, allowing us to isolate signatures of human-like performance. A large-scale, systematic search through feedforward neural architectures revealed that relatively shallow (three-layer) networks predicted human judgments better than any other models we tested. This is the first image-computable model that emulates human errors and succeeds in distinguishing mirror from glass, and hints that mid-level visual processing might be particularly important for the task.

## Introduction

Different materials, such as steel, silk, meat, or glass, have distinctive visual appearances, and our ability to recognize such materials by sight is crucial for many tasks, from selecting food to effective tool use. Yet, material perception is challenging. The retinal image of a given object is the result of complex interactions between the object's optical properties, three-dimensional shape, and the incoming light (Adelson, 2001; Fleming, 2014; Komatsu & Goda, 2018). Thus, a given material can take on an enormous variety of different appearances, depending on the lighting, object shape, and viewpoint. At the same time, similar objects with different material properties can create quite similar images in terms of the raw spatial patterns of color and intensity (Fleming, Dror, & Adelson, 2003). To succeed at material perception, the visual system must somehow tease apart similar images belonging to different materials, while at the same time grouping together very diverse images belonging to the same material class (Rajalingham et al., 2018; Storrs, Anderson, & Fleming, 2021; van Assen & Fleming, 2016). This is a fundamental aspect of biological visual processing, which remains poorly understood.

**Figure 1.**
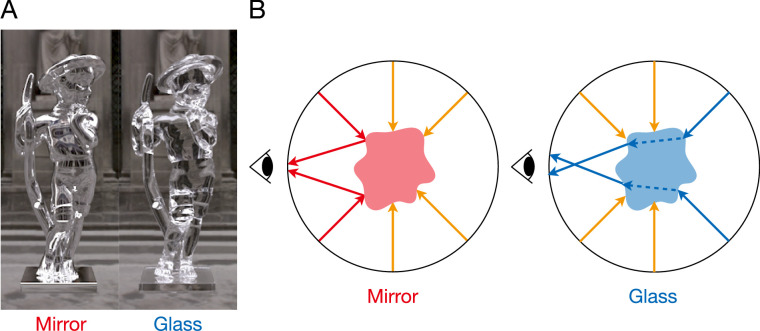
Distinguishing mirror from glass. (A) Example objects made of mirror (left) and glass (right) materials. The three-dimensional shape, illumination, and camera position are identical but the object's optical properties are different. (B) Illustration of different light paths through mirror and glass objects. Mirror reflects from the surface; glass refracts through the body of the object.

A particularly challenging case is to distinguish polished mirror-like specular materials (“mirror”) from colorless transparent materials (“glass”) (Fleming, Jäkel, & Maloney, 2011; Kim & Marlow, 2016; Schlüter & Faul, 2014, 2016; Tamura, Higashi, & Nakauchi, 2018; Tamura & Nakauchi, 2018). Both kinds of materials derive their appearance entirely from their surroundings, but through different light transport processes (Figure 1). Mirrors create a distorted reflection of the surrounding world, whereas, for glass materials, incident light also enters the material, refracts, and may reflect internally multiple times before re-emerging. Yet, in both cases, changing the object's shape or surrounding world radically alters the image. As a result, the visual cues we use to distinguish between mirror and glass must generalize well across an enormous variety of images. At the same time, to distinguish the two kinds of material, the visual system must presumably use quite sophisticated image measurements that latch onto subtle differences in the image resulting from the way light interacts with them. Thus, investigating how the brain distinguishes mirror from glass can tap into the core processes underlying visual surface appearance more generally.

We reasoned that, to work out how the visual system distinguishes mirror from glass, it is useful to take a big data approach, in which we embrace the enormous diversity of images of mirror and glass materials that confront the visual system. In particular, using computer graphics we sought to create a data set of hundreds of thousands of images, to encourage our human observers, and our model to use general purpose cues rather than image differences that apply only to a restricted, parametrically varying stimulus set. We used highly accurate state-of-the-art physics-based light-transport simulations to generate the images. To develop an image-computable model that distinguishes mirror from glass, we turned to deep learning methods.

In recent years, artificial neural networks (Cun et al., 1990; Lecun, Bottou, Bengio, & Haffner, 1998) have demonstrated significant potential as models of biological vision (e.g., Cichy, Khosla, Pantazis, Torralba, & Oliva, 2016; Guclu & van Gerven, 2015; Jozwik, Kriegeskorte, Storrs, & Mur, 2017; Khaligh-Razavi & Kriegeskorte, 2014; Kheradpisheh, Ghodrati, Ganjtabesh, & Masquelier, 2016b; Yamins et al., 2014; see also reviews from Kietzmann, McClure, & Kriegeskorte, 2019; Kriegeskorte & Douglas, 2018; LeCun, Bengio, & Hinton, 2015; Majaj & Pelli, 2018; and Yamins & DiCarlo, 2016). We set out to leverage these advances to approximate human visual processes underlying the challenging material perception task of distinguish mirror from glass. Comparisons between human vision and computational models typically use randomly selected images (Ghodrati, Farzmahdi, Rajaei, Ebrahimpour, & Khaligh-Razavi, 2014; Kheradpisheh, Ghodrati, Ganjtabesh, & Masquelier, 2016a, 2016b; Yamins et al., 2014), for which both humans and models achieve high performance. In contrast, our goal was to develop a model that could not only predict the successes of human judgments, but also systematic errors (Geirhos, Meding, & Wichmann, 2020; Golan, Raju, & Kriegeskorte, 2020; Macke & Wichmann, 2010), which are presumably the hallmarks of the processes unique to human visual computations. To do this, we created a diagnostic image set that yielded systematic and consistent visual errors as well as correct percepts. We show that, although conventional neural networks and hand-engineered models fail to predict the pattern of human judgments, neural networks with similar architectures exist that perform much more like humans. As an initial baseline measure of overall performance, we started by testing humans (Human experiment 1) and a variety of computational models on random our computer graphics data set (convolutional neural network [CNN] experiment 1). We then performed a sequence of lab and crowd-sourced experiments to identify a set of images in which human performance was systematically decoupled from ground truth (Human experiment 2). We then used this data set to select between thousands of neural networks, trained to distinguish mirror and glass, to identify those that behaved most similarly to humans (CNN experiment 2).

## Data set generation

**Figure 2.**
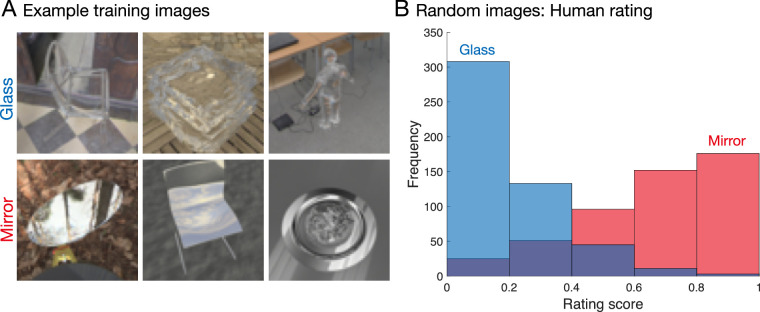
Random renderings by human observers. (A) Example renderings from the data set. *Top row*: glass; *bottom row*: mirror. Note that these example images were rated as glass and mirror, respectively, by all observers unanimously with a five-point scale (see Human experiment 1). (B) Results of rating experiment with randomly selected images. The horizontal axis indicates rating score from glass to mirror normalized between zero and one. The vertical axis indicates frequency of ratings in each bin across 10 observers. We treated values of less than 0.5 as glass and of 0.5 or greater as mirror. This criterion was also used for the classifiers (mentioned below).

Images were rendered using Mitsuba renderer (Jakob, 2010). We selected 1,583 objects from Evermotion (https://evermotion.org) and 253 high dynamic range light fields for the illumination from the Southampton-York Natural Scenes data set (Adams et al., 2016), the illumination data set (Debevec, 1998; Debevec et al., 2000), and other sources (see the Supplementary Information for complete list). To simulate different optical appearances for mirror and glass, we used Mitsuba's different bidirectional scattering distribution function models to capture the different ways that light interacts with surfaces and media. For the mirror objects, the bidirectional scattering distribution function was a conductor model with 100% specular reflectance. For the glass material, the bidirectional scattering distribution function was dielectric, with an internal refractive index of 1.5. Objects were uniformly scaled to fit within the unit sphere, and placed at the origin. The camera was randomly located at a position between 30° and 60° of elevation angle and any azimuth, with a constant distance of 2 units in Mitsuba. Mirror and glass images were generated in pairs using the same object and illumination but with different camera locations to avoid the classifiers simply learning a pixel difference between mirror and glass images. No explicit information was provided to humans or the models about which images belonged to the same pair. The sampling count was 512 per pixel with the Sobol Quasi-Monte Carlo sampler. The reconstruction filter was set as Gaussian. The renderer generated the final image, at 256 × 256 pixel resolution with gamma correction (Reinhard, Stark, Shirley, & Ferwerda, 2002). Then, they were resized to 64 × 64 to enable efficient training and test for the neural networks (see Figure 2A for examples). We screened all images and excluded a small number of images with rendering artifacts. The final data set contains 753,696 images, and is available for download from Zenodo [10.5281/zenodo.3229000].

## Human experiment 1: Random renderings by human observers

### Methods

#### Observers

Ten observers were students of Justus Liebig University Giessen with normal or corrected-to-normal vision (7 women; age range, 19–38 years; average 25.1 ± 5.2 years) in this experiment. All experimental protocols were approved by the Ethics board at Justus Liebig University Giessen and were conducted in accordance with the Code of Ethics of the World Medical Association (Declaration of Helsinki). Informed consent was obtained from all observers.

#### Apparatus

Stimuli were displayed on a 27-inch liquid crystal display (Eizo CG277) using factory default settings with a resolution of 1920 × 1200 pixels and a 60-Hz refresh rate. Stimulus presentation was controlled by MATLAB using Psychtoolbox 3.0 (Brainard, 1997; Kleiner et al., 2007; Pelli, 1997).

#### Stimuli

We randomly selected 1,000 images from the 64 × 64 pixel resolution data set (500 mirror and 500 glass images) as stimuli in Experiment 1. These were selected independently for the two classes (i.e., images were not paired with the same shape and lighting in both sets).

#### Procedure and task

Stimuli were presented in random order to each observer (i.e., each image was rated by all 10 observers). The images subtended a 2.2° visual angle. They were presented on a uniform gray background with a fixation cross in the center of the screen. The observers were asked to rate each stimulus on a 5-point scaling, where 1 indicated that the object looked compellingly like glass and 5 indicated that it looked compellingly like a polished metal, and intervening values indicated different degrees of ambiguous appearance (glass to mirror). We used the 5-point scale rating because 1) this provides more precise estimates on a per trial basis than a binary task, and 2) we compare more directly the rating score of the humans and models after normalization (Dropped). Responses were given by pressing the corresponding key on the keyboard. The observers could respond at any time, but the stimulus disappeared after 1 second. The experiment consisted of one session, approximately 30 minutes long, per participant including instructions. We did not explicitly include any breaks during the session, but they could take breaks anytime.

### Results and discussion

We first computed a frequency of the ratings averaged across all observers for each of the stimuli. Figure 2B shows a clear bimodal distribution of ratings, with mean ratings of 0.66 for mirror images and 0.19 for glass, and an average accuracy of 77.9 ± 3.3% standard deviation. This performance is comparable to the accuracy obtained with higher resolution stimuli (256 × 256) in a previous study (Tamura et al., 2018). Experiment 1, therefore, confirms that observers are capable of distinguishing mirror from glass for our data set. We next compared the human judgments with a variety of image-computable models to test whether they could also distinguish mirror from glass in our diverse computer graphics image set.

## CNN experiment 1: Random renderings by classifiers

Before investigating deep learning models in depth, we measured the extent to which relatively simple image measurements could predict perceptual mirror/glass judgments. The motivation for this was to test a number of controversial hypotheses about the kinds of features that may underlie material perception judgments (Anderson & Kim, 2009; Kim, Marlow, & Anderson, 2011; Motoyoshi, Nishida, Sharan, & Adelson, 2007; Sharan, Li, Motoyoshi, Nishida, & Adelson, 2008). Despite the similarities of mirror and glass, it might be possible to distinguish them reliably just using the raw marginal distributions of color values across pixels (e.g., if mirrors are on average higher contrast, or more strongly saturated than glass objects). Although there are strong grounds for doubting that humans only rely on such simple pixel histogram features (Anderson & Kim, 2009; Beck & Prazdny, 1981; Kim & Anderson, 2010; Todd, Norman, & Mingolla, 2004), this provides a useful baseline against which to compare less trivial models. Other work suggests that many aspects of perception may rely on mid-level texture-like image representations that capture joint image features across colors, scales, and orientations (Balas, Nakano, & Rosenholtz, 2009; Freeman & Simoncelli, 2011; Harvey & Smithson, 2021; Hiramatsu, Goda, & Komatsu, 2011; Rosenholtz, 2011). We, therefore, sought to test the extent to which such texture-like representations could predict human judgments of mirror versus glass.

### Methods

#### “Hand-engineered” and CNN classifiers

We developed three different classifiers (see Figure S1A for details): Color-Hist, Port-Sim, and a CNN with manually selected hyperparameters. All of the classifiers were presented with the entire image, including the background (i.e., the object of interest was not segmented from the background). The Color-Hist classifier used eight features: mean, variance, skewness, and kurtosis of intensity and color saturation from a 64 × 64 RGB image. The features of Color-Hist were z-scored across all images. To get the features of Port-Sim, we first used the texture analysis algorithm of Portilla and Simoncelli (Portilla & Simoncelli, 2000) to extract 3,381 higher-order image features. These were z-scored and the number of dimensions decreased to 1,052 by principal component analysis (cumulative explained variance of complete image set of >99%). For both the Color-Hist and the Port-Sim classifiers, a logistic regression was trained to distinguish mirror from glass based on the ground truth labels.

The CNN was defined as a three-layer convolutional neural network with 64 × 64 RGB image input and the binary (mirror vs. glass) classification output. The network architecture and training hyperparameters are shown in Figure S1B.

All classifiers were trained and tested with two-fold cross-validation, which was repeated 10 times with different randomly selected training and test sets. Each instance of the network was trained from a different initial random state. Note that the random images for human psychophysics (Figure 2B) were not used in this training. The final output—a prediction score ranging from 0 (as glass) to 1 (as mirror)—was averaged across training repetitions.

### Results and discussion

#### Classifiers’ performance

**Figure 3.**
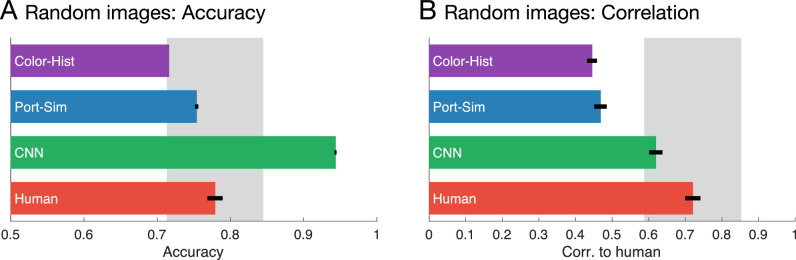
Results of randomly selected renderings. (A) Accuracy of human and model classifier responses for the test stimuli in experiment (mean of 10 repetitions in each classifier or 10 observers). Error bars represent standard error of the mean (error bar of Color-Hist is too small to see at this scale). Gray area indicates mean ± 2 SD of all human observers. (B) Correlation coefficient between human and model classifiers for the test stimuli. Human-to-human correlation was defined as the average of 10 correlations between each observer and the mean of the remaining observers.

Surprisingly, although based on quite simple image measurements, both the Color-Hist and Port-Sim classifiers achieved accuracies that almost rivaled human performance on the 1,000 randomly chosen images rated by our observers (Figure 3A). This finding suggests that, despite the complex optics of reflection and refraction, there are many potential cues that would suffice to perform significantly above chance at distinguishing the two kinds of materials. A more precise test of how well such cues emulate human judgments is the correlation between the Color-Hist and Port-Sim models and humans on an image-by-image basis. Although the models did correlate significantly with human performance, they did so significantly less well than individual humans do, Color-Hist versus humans: *t*(18) = 6.056, *p* < 0.001; Port-Sim vs humans: *t*(18) = 2.356, *p* < 0.05, *t*-test, suggesting that humans do not rely on the same cues as these simple classifiers (Figure 3B).

On the same random images as before, the CNNs achieved an accuracy that superseded humans and correlated with mean human responses within the same range as individual humans did, thus outperforming the two hand-engineered classifiers (Figures 3A and 3B). This finding suggests that CNNs learn features that are inherently superior to the simple color and texture features.

This finding in itself is unsurprising, because the CNNs learn many more features (99,410), and thus perform the classification in a higher-dimensional space. However, for the purposes of understanding biological vision, the key question is whether the features learnt by the CNNs lead to performance that more closely resembles human judgments on an image-by-image basis.

#### Representational similarity analysis

To gain further insights into the nature of the internal representations of the classifiers, we performed representational similarity analysis (Kriegeskorte, Mur, & Bandettini, 2008) using the images that had been rated by humans. We defined two different representational dissimilarity matrices (RDM), a first-stage RDM to identify dissimilarity relationships between images in humans and each classifier; and a second-stage RDM (classifier dissimilarity matrix), characterizing how similar the first-stage RDMs are across different humans and classifiers, allowing us to compare their internal representations. The first-stage RDM was defined as Euclidean distance of prediction scores (final output) from each classifier or average of observers’ response from human. The second-stage RDM was defined as a dissimilarity, which was one minus Pearson's correlation between each first-stage RDMs. Note that each entry in the first-stage RDM is synonymous with a subtraction between two scalar values. We use the expression RDM to maintain consistency.

**Figure 4.**
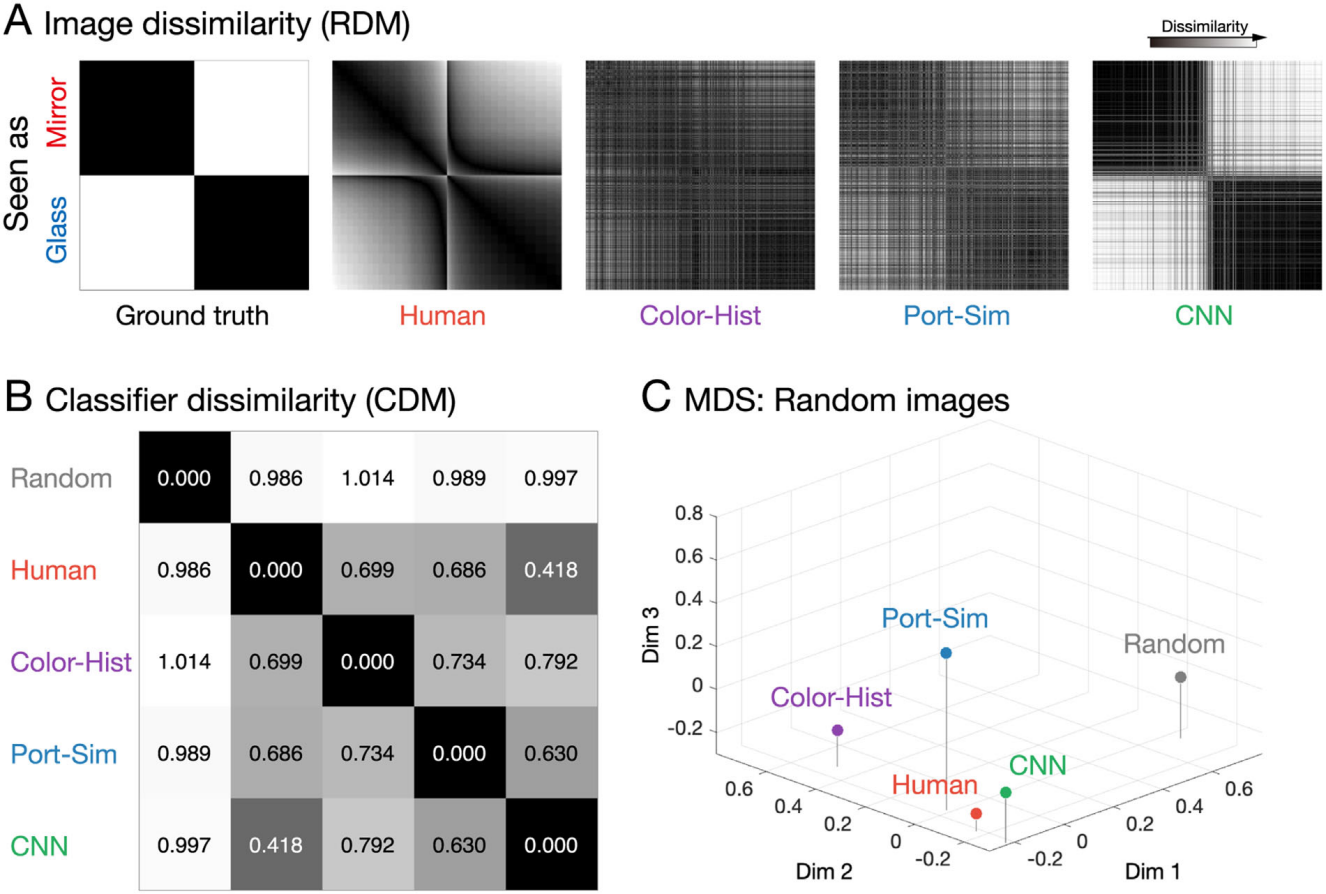
Representational similarity analysis (RSA) of randomly selected renderings. (A) RDMs for ground truth, each classifier and human judgments. The rows/columns of each matrix represent 1,000 images, ordered into two blocks by their true class (mirror vs. glass) and within a block by their mean human ratings from most mirror-like to most glass-like. Individual entries represent the dissimilarity between the corresponding pair of images in terms of the perceived or predicted mirror vs. glass ratings. Darker entries indicate that the corresponding images are estimated to be highly similar, whereas brighter entries indicate they are more dissimilar. Note that the blocks corresponding to mirror include some images that humans incorrectly classify as glass and vice versa. (B) A classifier dissimilarity matrix (CDM) between models/humans. Each row/column indicates a different classifier, human, and random RDM as a control. Each entry contains the mean correlation distance (1 – correlation) between the RDMs for the corresponding pair of observers/models (intensity code as in A). (C) A three-dimensional visualization of the relationship between models/humans by applying multidimensional scaling (MDS) to the CDM. The three axes indicate the first three dimensions obtained by MDS with 95% cumulative explained variance.

Figure 4A shows the first-stage RDMs for each of the classifiers, as well as ground truth. The rows/columns of the matrix represent the different images, ordered into two blocks by their true class (mirror vs. glass) and within a block by their mean human ratings (from most mirror-like to most glass-like). Individual entries represent the dissimilarity between the corresponding pair of images in terms of the perceived or predicted mirror versus glass ratings. Thus, low values indicate the corresponding pair of images are represented as highly similar, while higher values indicate they are more dissimilar. The patterns in the matrices suggest that, for these randomly selected images, humans and classifiers broadly agree.

We can summarize the relationships between the RDMs in second-stage RDMs (classifier dissimilarity matrix; see Figure 4B), in which each row/column indicates a different observer or computational classifier, and each entry contains the mean correlation distance (1 – correlation) between the RDMs for the corresponding pair of observers/models (Figure 4B). For comparison, we also included 10 random RDMs, to characterize how much more dissimilar the classifiers are to humans than would occur by chance. The dissimilarity between each classifier and humans were 0.699 (Color-Hist), 0.686 (Port-Sim), and 0.418 (CNN), respectively, suggesting that the Color-Hist and Port-Sim use more different representations than the CNN to humans.

Applying multidimensional scaling to the classifier dissimilarity matrix allows us to visualize the relationships in three dimensions (Figure 4C). This process reveals that all three classifier types learn interimage relations that are significantly and substantially closer to humans than occurs by chance (Random), and of all classifier types, the CNNs seem to acquire the most similar representation to humans. These results tend to suggest that feedforward convolution neural networks have significant potential as models of human visual judgments of mirror and glass materials.

## Human experiment 2: Creating a data set of images diagnostic of human vision

Based on the significant correlations between observers and the computational models, it could be tempting to conclude that the models simulate human visual processes. However, there are several reasons for caution. First, the main purpose of comparing models based on different features is to identify which features best predict human material perception. Yet, for randomly selected images, even the most primitive models appear to match human perception quite well. Given what we know about early vision and material perception (Anderson & Kim, 2009; Kim & Anderson, 2010; Marlow, Kim, & Anderson, 2012), it seems highly unlikely that visual perception of mirror versus glass is based on raw luminance and color distributions, which are entirely insensitive to the spatial structure of the image. Second, and more important, it is possible that the high correlations simply result from the fact that both humans and classifiers achieve quite high accuracies. If all models correctly assign most images to one of the two distinct modes (mirror or glass), then it follows that they will tend to correlate with one another. Indeed, in Figure 4A, 58% of the variance in the human judgments is accounted for by the ground truth. A good model should be able to predict not only the successes of human vision, but also the specific pattern of errors, on an image-by-image basis. To test this, we sought a set of diagnostic images that decouples accuracy from human judgments.

**Figure 5.**
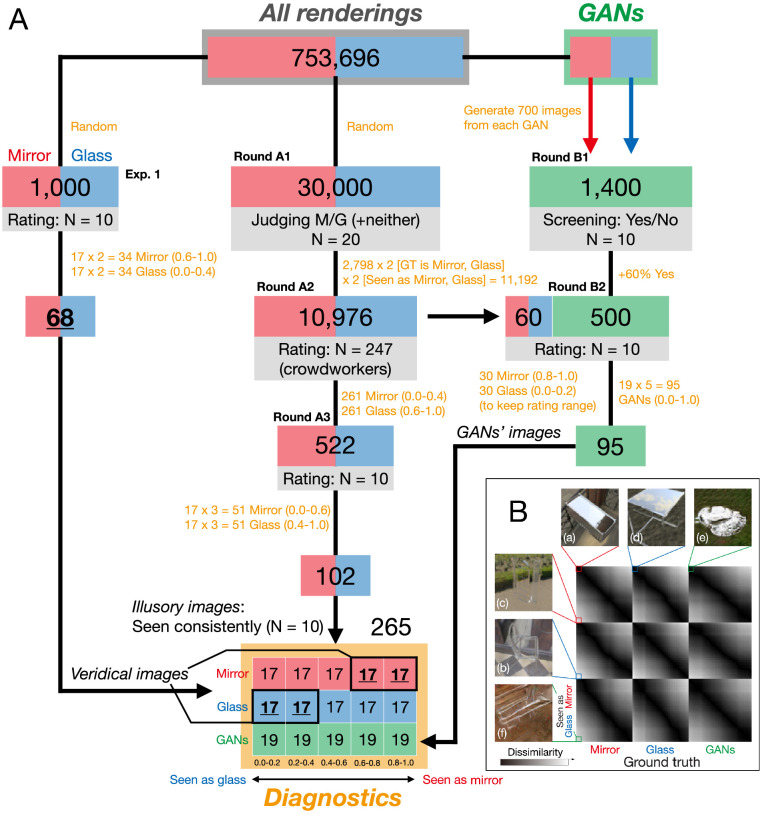
Creating a data set of images diagnostic of human vision. (A) Flowchart describing creation of diagnostic image set, which consists of three different image types: “veridical” renderings (human judgments match ground truth), “illusory” renderings (humans consistently misjudge material class), and “GAN” images. Red indicates renderings depicting mirror materials; blue indicates renderings of glass materials; green indicates images generated using GANs. Each stage represents a different experiment used to select images for the subsequent stage in the corresponding sequence (Round A and B). See Methods for details of selection process. (B) RDM of diagnostic image set. The format is the same as Figure 4A except adding GANs as the third class. The panel shows six example images (a–f) with extremely high or low rating score in each class.

Creating such a data set is nontrivial; most images are perceived correctly. It is not sufficient to identify images for which participants are inconsistent in their interpretation, because a deterministic, image-computable model cannot even in principle account for variations between observers when presented with the same image. Our goal is to predict that proportion of the variance in judgments that is consistent across observers; therefore, we need an image set that includes images that are consistently misperceived. Specifically, the goal was to identify a benchmark set of images in which each image was consistently judged across observers, but across the data set as a whole, there was a flat distribution across the five bins ranging from mirror to glass ratings, for both mirror and glass images (in contrast with the skewed distributions for random images in Figure 2B). Such a data set would thereby decorrelate the true material class from the perceived class. We set as our criterion of consistency that 10 of 10 naïve observers should rate each image in the same bin of the 5-point rating scale. To create the diagnostic image set, we used a series of laboratory and crowdsourcing experiments to filter through a large number of images (see Figure 5A for an overview).

### Methods

#### Observers

##### Lab experiments (i.e., not crowdsourcing)

Fifty observers were students of Justus Liebig University Giessen and Toyohashi University of Technology with normal or corrected-to-normal vision (see *Procedure and Task* for details). All experimental protocols were the same as in Experiment 1.

##### Crowdsourcing

We recruited 247 participants were recruited via the Clickworker platform and were paid 1.2 Euro each. Before the beginning of the experiment, participants were presented with an online consent form that explained the purpose and procedure of the experiment, as well as the uses and benefits of their participation. All participants that took part in the experiment agreed to these conditions and that their data be recorded and stored anonymously for research and publication in scientific journals.

#### Apparatus

The apparatus for the laboratory experiment was the same as in Experiment 1, except using the LCD (Eizo CG276 at Toyohashi University of Technology and CG277 at Justus Liebig University Giessen).

#### Stimuli

##### Renderings

We randomly selected another 30,000 images from the data set and started a series of experiment (series A1–3; central path in Figure 5A). Note that these new images did not include the 1,000 images already set aside as the random set.

##### GAN images

Because rendering were rarely misclassified, we sought a method to increase the number of diagnostic images. To do this, we trained a generative adversarial network (GAN; Goodfellow et al., 2014; Radford, Metz, & Chintala, 2015) on our renderings. GANs consist of a generator network, G, that is trained to produce images, which a discriminator network, D, has to distinguish from a given data set. During training, D improves at distinguishing the synthesized images from the training data, and G learns to create images that are hard to discriminate from the training data. In this study, two GANs were trained to synthesize images based on mirror and glass training sets, which includes all 376,848 images in each class from the data set. Once trained, new images could be synthesized by feeding G random input vectors. We defined images produced by the network trained on mirror images as having mirror ground-truth label, and likewise images produced by the network trained on glass images as having glass ground-truth label. However, the resulting images frequently had a more ambiguous, intermediate appearance than renderings did, leading to a greater number of misclassifications and allowing us to increase the number of images in the diagnostic set. The network architecture and hyperparameters were the same as a previous network (Radford et al., 2015), except for minor modifications in the standard Tensorflow DCGAN implementation that avoid the discriminator network converging too fast (Kim, 2017). After 20 epochs training, we then generated 700 images from each GAN by inputting random noise vectors to create a total of 1,400 images, which were then rated by humans (see right-hand path in Figure 5A).

#### Procedure and task

The purpose of this experiment was to create a benchmark set of diagnostic images, with 1) a uniform distribution of appearances ranging from mirror to glass, 2) perceptual appearance that is decorrelated from the true material class (ground truth), and 3) consistent judgments across observers. Identifying images that positively correlate with ground truth (example image (a) and (b) in Figure 5B) is straightforward, as humans are generally good at distinguishing mirror versus glass for our renderings. Thus, most of the procedure revolves around finding images that systematically yield errors (i.e., example image (c) and (d) in Figure 5B). To achieve this, we ran two parallel series of experiments using renderings (series A) and images generated by GANs (series B), respectively. Each series starts with a large number of images, with images being progressively excluded in each round, to arrive at a much smaller final set covering the desired distribution (see also Figure 5A).

##### Round A1 (rendering ratings)

Twenty observers (all men; age range, 21–26 years; average 23.1 ± 1.4 years) participated in the laboratory. We randomly selected 30,000 renderings from the data set (50% mirror, 50% glass) and distributed 1,500 images to each observer. The procedure was the same as in Experiment 1, except that the task was a three-way judgment (mirror, glass, or hard to recognize). The observers were instructed to use the last option if it was difficult to recognize the stimulus. Only 2.9% of the images fitted into this and they were excluded to avoid contaminating rendering artifacts for further rounds here. Figure S2A shows results of this round. In total, 10,976 images moved ahead to round A2. Specifically, 2,744 images were selected randomly from each of the four bins other than the hard to recognize images (i.e., mirror that looks like either mirror or glass and glass that looks like either mirror or glass).

##### Round A2 (rendering ratings)

Next, 247 crowdsourced participants observed the stimuli selected by round A1 and were asked to rate them on a 5-point scale (glass to mirror). They were each shown 100 images—98 randomly chosen test images from the output of round A1 and two catch trial images, consisting of photographs with a clear mirror or glass appearance. If they responded incorrectly in either of these catch trial images, the participant was rejected for further rounds. Only the 5,586 images that were rated by at least three crowd-workers were analyzed further. Figure S2B shows rating results of this round. Based on the responses, we selected 522 images, in which the ratings conflicted with the ground truth material, by selecting from the two outermost bins of the distribution for each class. Specifically, 261 mirror images with a rating score of 0.0–0.4 (i.e., seen as glass) and 261 glass images with a rating score of 0.6–1.0 (i.e., seen as mirror). These images progressed to round A3.

##### Round A3 (Rendering ratings)

Ten observers participated in the laboratory (9 women; age range, 21–30 years; average 24.8 ± 2.8 years). The procedure was the same as in Experiment 1 (a five-bin rating task). The experiment consisted of 1,566 trials (three trials × 522 images from round A2), and all trials were randomly ordered. Figure S2C shows results of this round. From these, a total 102 images were selected for the diagnostic image set, by selecting from the three outermost bins from each class. Specifically, 51 mirror images with ratings of 0.0–0.6 (i.e., seen as glass or ambiguous) and 51 glass images with ratings of 0.4–1.0 (i.e., seen as mirror or ambiguous). These selected images were included in the diagnostic image set.

##### Round B1 (GAN image screening)

Some GAN-generated images resemble textures rather than objects with distinct material properties. The purpose of Round B1 was to exclude such images from subsequent rounds. Ten observers participated in the lab (8 women; age range, 20–32 years; average 24.8 ± 4.1 years). The stimuli were 1,400 images generated by GANs (see *GAN images*). The procedure was the same as in Experiment 1, except that the task was to indicate in a binary decision whether the object shape and material were recognizable or not. Figure S3A shows results of this round. Based on the responses, 500 images that were judged to be recognizable by at least 6 out of 10 observers were moved ahead to Round B2.

##### Round B2 (GAN image ratings)

Ten observers participated in the laboratory (all women; age range, 21–34 years; average 25.1 ± 3.8 years). The stimuli were 560 images including 500 GAN images from Round B1 and 60 renderings (30 mirror and 30 glass images) from round A2, which had received ratings that were highly consistent with ground truth. The procedure was the same as in Experiment 1 (rating task). The experiment was composed of 1,680 trials (3 trial × 560 images from Rounds B1 and A2), and all trials were randomly ordered. Figure S3B shows result of this round. We selected 95 images (19 images from each bin) to add to the diagnostic image set in order to satisfy the flat distribution for the GAN images in the same manner as for the mirror and glass renderings.

##### Final diagnostic image set

**Figure 6.**
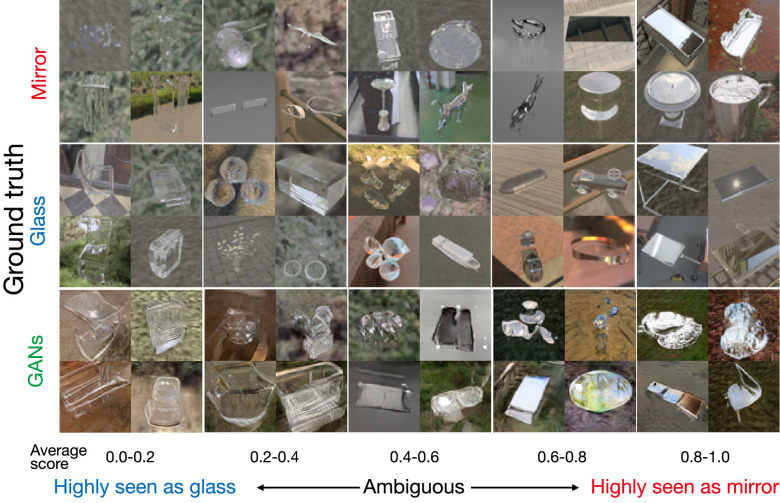
Example images from the diagnostic image set. Each row indicates different ground truth (mirror, glass, and GANs). Each column indicates average rating score of 10 observers.

The two streams of experiments resulted in a final diagnostic image set of 265 images including both mirror and glass renderings, along with GAN images with prediction score uniformly distributed from 0.0 to 1.0 (Figure 5B and Figure 6). These are composed of 68 veridical images (from Experiment 1), 102 illusory images (from Rounds A1 to A3), and 95 GAN images (from Rounds B1 and B2) (see Figure 5A in detail).

### Results and discussion

From the responses in rounds A1–A3, we ended up with a set of 170 renderings, that is, 34 images in each of the 5 bins from perceived mirror to perceived glass, one-half of which were actually mirror and one-half glass. This practice allowed us to decorrelate images consistently seen as one category by humans from the true category of the images.

To increase the number of images for further use, we also trained two GANs to synthesize images with many of the visual characteristics of the renderings from each class. We find that such images include many cases that are more ambiguous than renderings, appearing somewhere between mirror and glass. In rounds B1 and B2, we identified 95 images (out a set of 1,400), which were consistently rated by 10 observers as belonging to specific bins. Combining the selected GAN images with the renderings yielded a total 265 diagnostic images, in which human judgments were perfectly decorrelated from ground truth that, for the GAN images, was defined by the class of images the GANs were trained on (Figure 5B). Some examples are shown in Figure 6. Interestingly, although it is possible to distinguish GAN images from renderings, those in the diagnostic image set (the bottom row in Figure 6) tend to seem to have a coherent object shape and appearance, somewhat similar to the renderings. This approach is not without limitations. All models have inductive biases, including GANs, and the fact that observers can distinguish between GAN images and rendering implies that they may contain cues that renderings do not, or lack cues that renderings possess. Nevertheless, our goal is not to model ideal material discrimination, but rather human judgments. By selecting images that 10 out of 10 observers agreed upon, we can ensure that the GAN images considered here yield consistent subjective impressions along the mirror–glass continuum even if they are not physically accurate renditions of objects. Although 265 images in total is small relative to the set of possible images of mirror or glass, these images are not used for training models, only for testing them.

## CNN experiment 2: Systematic exploration of the space of feedforward networks

**Figure 7.**
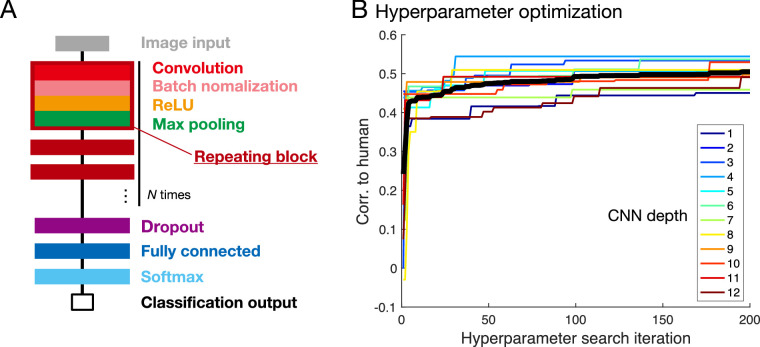
Development of OptCNN. (A) General form of the feedforward network architecture in this study (see also Figure S1B). (B) Results of the Bayesian hyperparameter search (BHS). The horizontal axis indicates the number of iterations of the BHS. The vertical axis indicates correlation between human to each model with different network depth (indicated by color, from 1 to 12). Thick black line represents mean of all 12 depths.

With the diagnostic image set in hand, we next sought neural network models that would positively correlate strongly with humans for these diagnostic images. It is important to note that the space of potential convolution network models is very large: they can vary widely in terms of their architectures, hyperparameters and training schedules. We reasoned that within the space of feedforward neural networks, some networks are likely to approximate human visual processing better than others. We therefore ran a large-scale search through a space of feedforward networks with the general form depicted in Figure 7A, varying the network depth systematically (see also Figure S4A).

### Methods

#### Network architecture

All networks consisted of an input layer followed by a basic block of layers composed of convolution, batch normalization (Ioffe & Szegedy, 2015), ReLU (Glorot, Bordes, & Bengio, 2011), and max pooling layers, which were repeated several times, followed by dropout (Srivastava, Hinton, Krizhevsky, Sutskever, & Salakhutdinov, 2014), fully connected and softmax layers, and ending with a two-unit classification output (mirror vs. glass); see Figure 7A.

#### Identifying optimal CNN models through Bayesian hyperparameter search

We used a Bayesian hyperparameter search through a space of feedforward architectures to identify which CNNs correlated best with humans (using MATLAB R2017b with Neural Network Toolbox and Statistics and Machine Learning Toolbox) (see Figure S4A). The objective was to maximize the correlation coefficient between CNN and human on the diagnostic image set. The network architectures were basically the same as the CNN in Experiment 1, except that we parametrically varied the depth, that is, the number of layers from 1 to 12, by repeating a block of layers consisting of convolution, batch normalization, rectified linear unit, and max pooling layers before the first fully connected layer (Figure 7A). Note that the maximum pooling layers were only used up to three layers (the last three layers) because of the size constraints of the filters.

For each depth, we ran 200 iterations of the Bayesian hyperparameter search to identify the values of 11 hyperparameters controlling the network architecture and training (e.g., number of filters per layer, initial learning rate, momentum; see Figure S4B) in an optimization stage (i.e., 200 CNNs were generated with different hyperparameters, in search of the optimal values for each depth). Each CNN was trained and tested with two-fold cross-validation using same training and test set, in which randomly chosen 400,000 renderings (200,000 images in each class) to converge the network quickly. Note that we did not use the images from the random set (1,000 images) for training the OptCNN.

Having identified promising hyperparameters for each architecture depth, in the validation stage, we then trained 30 instances of each of the resulting neural networks (differing only in the initial random state), again using the same number of renderings, with half for training and the other half for testing (see Figure S4B). Importantly, these networks were never trained on the diagnostic images, and training proceeded until the validation accuracy had not improved for at least three validations, independently for each architecture depth.

### Results and discussion

**Figure 8.**
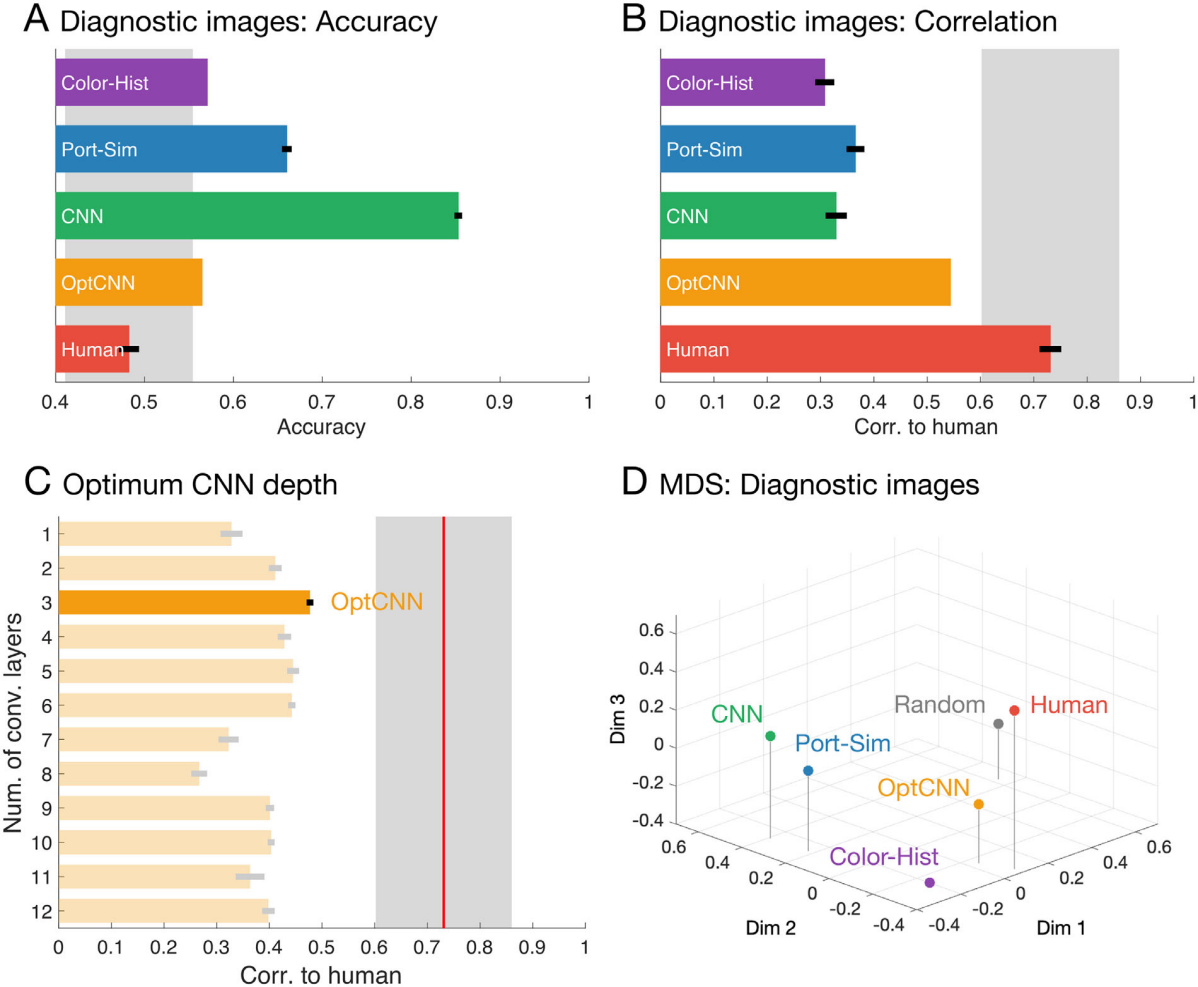
Results of the diagnostic image set. (A) Accuracy of humans and model classifiers for diagnostic image set. (B) Correlation coefficient between human and classifiers for diagnostic stimuli (symbols are the same as in Figures 2A and 2B). OptCNN represents the highest correlation of 30 instances of three-layer CNN from Bayesian hyperparameter search. (C) Correlation to humans for each network depth. The horizontal axis indicates correlation coefficient. The vertical axis indicates the number of convolution layers (i.e., the number of repeating blocks) in the networks. Red line and gray area indicate mean ± 2 SD of all human observers. (D) A three-dimensional visualization of the relationship between the models via multidimensional scaling (MDS) with 73% cumulative explained variance (as in Figure 4C, but based on diagnostic image set).

The mean correlations between the networks and humans on the diagnostic image set is shown in Figure 8C. Of all depths, the three-layer network architecture was the one that correlated best with human judgments, which we refer to as optimal CNN (OptCNN), although this does not imply that it represents a global optimum across all possible neural networks, only the best of those we tested. The layer depth of OptCNN was relatively shallow even though we systematically searched through 1- to 12-layer networks. We considered this network class for further analysis.

Figures 8A and 8B compare the highest of the OptCNN networks with humans and the other classifiers on the diagnostic image set. By design, humans perform at chance on these images (Figure 8A). All classifiers outperform humans in terms of accuracy, yet OptCNN is the closest, making the most errors on these images, even though it was trained with the same objective function and a very similar training set to the original CNN, which performs too well to resemble humans. In terms of image-by-image correlations to human judgments (Figure 8B), none of the classifiers reaches the noise ceiling, but OptCNN significantly outperformed all other classifiers, Color-Hist versus OptCNN: *t*(9) = 4.79 × 10^13^, *p* < 0.001; Port-Sim vs OptCNN: *t*(9) = 5.19, *p* < 0.001; CNN versus OptCNN: *t*(9) = 20.23, *p* < 0.001, *t* test.

However, despite OptCNN behaving a lot more like humans than any of the other models, it is still not a perfect model. Indeed, none of the thousands of networks trained throughout the Bayesian hyperparameter search or the final validation exceeded a correlation with humans of 0.54 whereas human-to-human correlations was between 0.61 and 0.81. In other words, although the OptCNN was the closest of all models we considered, it still failed to capture average human performance as well as even the most unrepresentative of the individual humans did. This finding suggests that more caution should be taken in comparisons between artificial neural networks and biological neural systems. Previous work has used much slacker criteria for the comparison and may have drawn erroneously positive conclusions.

The performance of OptCNN for the random set was 72% accuracy and 0.45 correlation to humans on average. This accuracy reached the human noise ceiling but the correlation did not, suggesting that while OptCNN avoided an overperformance like the CNN, it still failed to match human judgments on arbitrary images.

Figure 8C shows the correlations between humans and the CNNs with different numbers of convolution layers. To the extent that OptCNN approximates human visual processes, the finding that it has only three layers tends to suggest that distinguishing mirror from glass does not involve as many stages of processing as object recognition, for example, which typically requires seven or more layers to approximate human performance (Jozwik et al., 2017; Kheradpisheh et al., 2016b; Kubilius, Bracci, & op de Beeck, 2016). It suggests the particular involvement of mid-level computations, one or two stages beyond simple local filter representations.

To compare the nature of the representations in the different classifiers and humans in greater detail, we performed a representational similarity analysis. To do this, we measured the similarity between all images in the diagnostic image set according to the final classification output of each classifier. To visualize the relationships between the different classifiers, we computed the mean correlation dissimilarity between the different classifier types, and then performed multidimensional scaling (as in Figure 4C). Figure 8D shows the different classifier types arranged in the first three resulting dimensions. This analysis reveals that OptCNN was the most similar to humans, although there remains a substantial residual difference.

**Figure 9.**
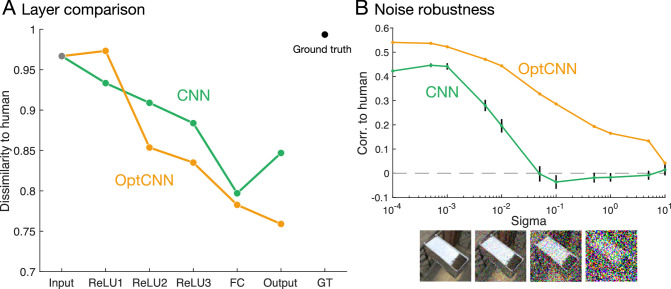
Comparing CNN and OptCNN in terms of dissimilarity to human and robustness to noise. (A) Dissimilarity to humans at each layer in CNN and OptCNN. The horizontal axis indicates the network layer from input to output, along with the ground truth (rightmost point), and the vertical axis indicates dissimilarity to humans. (B) Robustness to noise. The horizontal axis indicates sigma of Gaussian noise (i.e., the amount of image perturbation). Four images show examples with different sigma (10^−3^, 10^−2^, 10^−1^, and 10^0^). The vertical axis indicates the correlation to humans. Error bars represent standard error of the mean across all 10 classifiers (CNN). Note that OptCNN represents the highest correlation of 30 instances (same as OptCNN in A).

The greater similarity between OptCNN and the default CNN is also revealed by a more detailed view into the representations at different processing stages of the networks. We applied a representational similarity analysis using the diagnostic image set, to the input stage, the three ReLU stages after each convolution layer, the fully connected layer and the final output in both the original CNN and OptCNN. We then computed a dissimilarity to humans as 1 – correlations between human performance and each of the resulting RDMs (layer correlation matrix). Figure 9A shows how this dissimilarity varies as a function of layer for CNN and OptCNN (as well as ground truth for comparison). At the input stages of the network, images that are perceived by humans as mirror and glass materials are thoroughly entangled, such that further processing is required to separate them. As we proceed through network layers, we see that the dissimilarity to human in CNN and OptCNN both decrease. However, from ReLU2 onward, all layers of the OptCNN have more similar representations to humans than the corresponding layer in CNN. It is interesting to note that the dissimilarity in the original CNN's fully connected layer is actually closer to humans than its final output. In other words, a read-out from the late and intermediate stages of the original CNN could also approximate human performance. At this stage of the network, glass and mirror are still substantially entangled—it is only in the final output that the network applies the last transformations that deliver high objective accuracy. Paradoxically, this increase in accuracy is associated also with a decrease in similarity to humans. In contrast, OptCNN's representations continuously approximate human judgments more closely with each layer. This finding suggests that human judgments are not optimal given the data available in the images for distinguishing mirror from glass. Instead, they resemble incompletely disentangled representations (as found in CNN's fully connected layer or OptCNNs output layer). This finding further reinforces the idea that general purpose mid-level image measurements—not specifically optimized for distinguishing mirror from glass—underlie human judgments.

Robustness to noise is another key characteristic of human vision (Geirhos et al., 2017). We find that the OptCNN outperforms the original CNN in terms of the effects of noise on the correlation with human performance. If we perturb the input images with noise, the networks’ predictions about the material tend to change (Figure 9B). Importantly, we find that while the correlation between CNN's predictions and the human judgments of the unperturbed images falls precipitously as noise is added, for OptCNN, not only is the correlation higher across all noise levels, the decline is also gentler. Our findings also suggests that, by identifying networks that more closely resemble humans in terms of their solution to the objective function, we also identify representations that capture other aspects of human perception, such as robustness to noise. Interestingly, previous work has shown that human discrimination of mirror versus glass is surprisingly robust across certain kinds of atypical viewing conditions, such as when the illumination consists of binary noise (Tamura et al., 2018). Under these conditions, the human visual system can nevertheless extract meaningful material percepts from other cues (e.g., motion).

**Figure 10.**
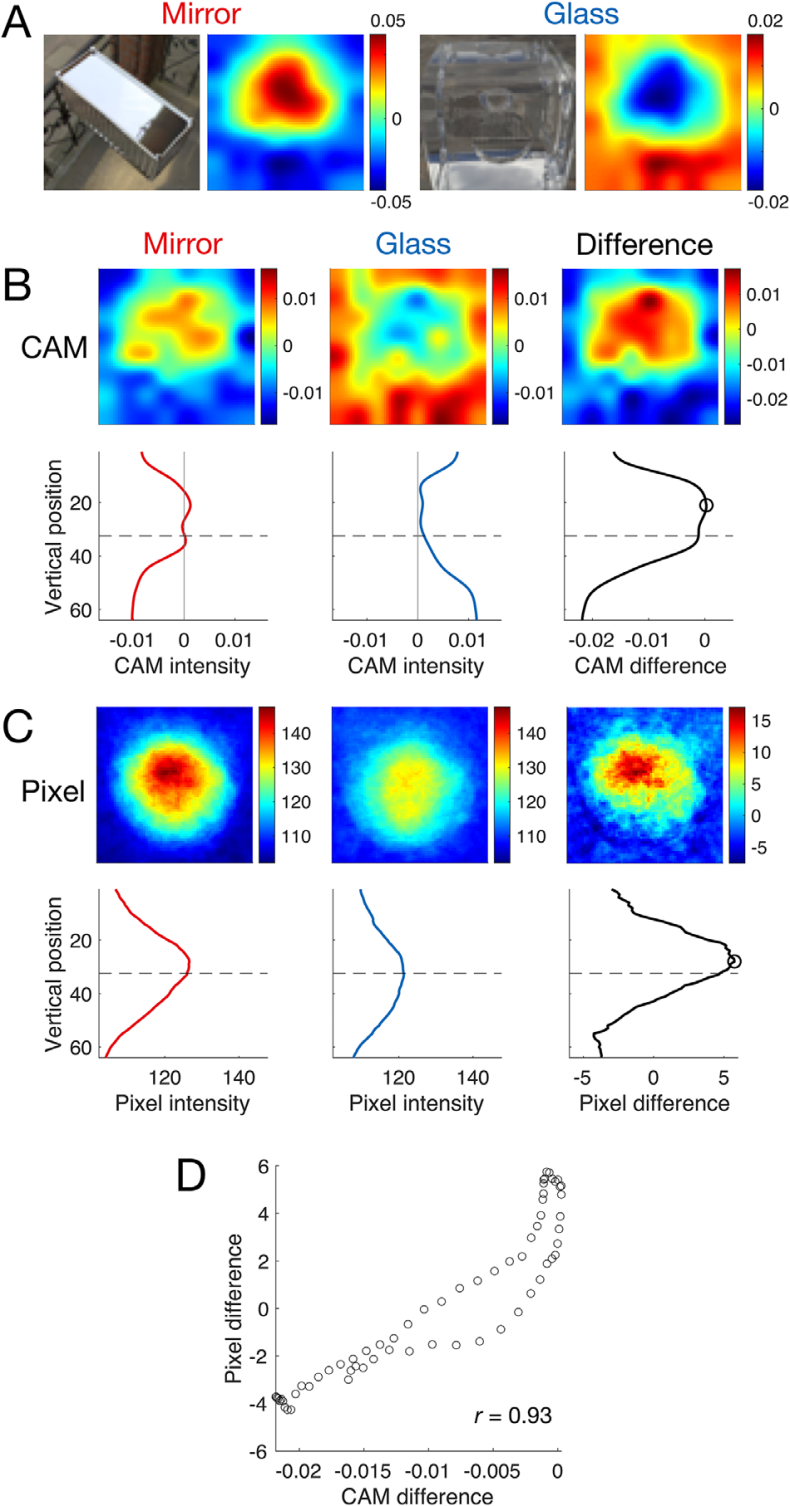
Class activation maps (CAM) and pixel intensity reveal systematic differences between mirror and glass images along the vertical direction. (A) Example CAMs for an individual mirror image (left) and glass image (right). (B) Top row: Mean CAM across all mirror images (left) and glass images (middle) from the ‘random’ set, along with the difference between the mean CAMs for the two classes (right). Bottom row: mean CAM intensity along the vertical direction for the corresponding CAM. The dashed line indicates the center of the image in the vertical direction. The circle in the bottom right graph indicates the peak of the CAM difference. (C) Same as B, except for pixel intensity instead of CAM. (D) Correlation between the CAM difference (the bottom right of B) and the pixel difference (the bottom right of C). They were significantly correlated (*r* = 0.93, *p* < 0.001).

Finally, we examined whether any of the features learnt by the OptCNN resemble those used by humans. To do this, we performed an analysis into the image features that drive the OptCNN network's decisions. We visualized activations of units in OptCNN, using class activation mapping (Zhou, Khosla, Lapedriza, Oliva, & Torralba, 2016). This method identifies which image locations are most responsible for the activation of mirror and glass units—here from a stage before the softmax layer (see example class activation maps for specific images in Figure 10A). Applying this method to all images in the random set revealed an intriguing regularity. We found systematically stronger activity in the upper one-half of the image driving mirror classifications, and stronger activity in the lower one-half of the image driving glass classifications (Figure 10B). This is most clearly indicated when we subtract the average class activation mapping for glass from that of mirror, to reveal the differences that drive the network's decisions (see Figure 10B right column; note the peak in the graph above the midline). The analysis suggests that the network is sensitive to a systematic spatial difference (along the vertical direction) between mirror and glass images in our data set. This prompted us to test whether such vertical bias was observed in the pixel intensity of the images (Figure 10C). We found that vertical variations in activation and average pixel intensities indeed correlated strongly (*r* = 0.93; Figure 10D).

The regularity in the images presumably results from the following environmental and physical considerations: 1) in natural illumination light comes more strongly from above than below; 2) convex mirror-like objects tend to have upwards-facing surface normals in the upper one-half of the image, reflecting the brighter sky; and 3) in contrast, convex refractive objects tend to invert the image, leading to greater brightness in the lower one-half of the image.

If this regularity influences network decisions, then presenting upside-down (vertically flipped), images should decrease network accuracy. As predicted, when we tested OptCNN with upside-down images, the overall classification accuracy decreased by 7% in the random set and 6% in the diagnostic set. Interestingly, we had previously found that humans exhibit a qualitatively similar decrease in performance with inverted images (Tamura et al., 2018). Thus, we suggest that the models depend on the vertical composition of the images in a similar way to humans.

## General discussion

Because many materials that we can easily recognize did not exist until the last few centuries—or even decades—our ability to recognize them must be learned rather than evolved. How the visual system acquires the visual computations and internal representations that allow us to succeed at material perception remains poorly understood. Here, we investigated the extent to which supervised deep learning can reveal representations that resemble human judgments.

Studies comparing humans with machine learning models often focus on overall performance at a task or correlation on arbitrary images (Ghodrati et al., 2014; Hong, Yamins, Majaj, & DiCarlo, 2016; Kheradpisheh et al., 2016a, 2016b; Kubilius et al., 2016; Majaj, Hong, Solomon, & DiCarlo, 2015) for which both humans and successful machine vision system tend to perform well. Here, by contrast, we applied a different criterion for comparing neural networks with human judgments, by creating a diagnostic set of images in which human performance is decorrelated from the ground truth. This process allows us to search for networks that capture the characteristic eccentricities of human vision, reproducing the tell-tale errors that humans tend to make as a first step toward identifying the specific cues and processes the human visual system uses. Although identifying such an image set is time consuming and effortful, it provides a benchmark against which all future models of human vision can be tested. Here, we created one such set for a challenging material perception task: the discrimination of mirror and glass materials.

We suggest that variance in performance across images can broadly be divided into two classes. First, there is variance that is strictly stimulus related, such that practically any successful classifier would be susceptible to it. For example, in the limit it is possible to make images of glass and mirror objects that are pixel-for-pixel identical by carefully tweaking illumination and shape (or, for example, choose conditions that yield images that are entirely uniform). No classifier could in principle distinguish degenerate cases such as these. But even where there are detectable differences between images, there are still some images that more strongly or reliably indicate the class than others. For example, when glass surfaces exhibit total internal reflection, the resulting patterns may be highly similar to those that would be created by a mirrored surface. This source of variance, which is likely to be substantial, may be useful for defining an ideal observer model, but does little to help us distinguish between models. It is also likely a subset of this variance that the Color-Hist and Port-Sim classifiers captured: essentially, projecting onto arbitrary features can enable at least some statistical success at distinguishing mirror from glass. One potential feature driving the success of the simple classifiers could be statistical aspects of natural illumination (Adams, Kucukoglu, Landy, & Mantiuk, 2018). For example, mirror objects in our data set tend to reflect more of the sky, leading to slightly higher average luminance and contrast than glass. Interestingly, humans also seem to exploit some of these differences. When illumination colors are inverted, human accuracy for distinguishing mirror from glass decreases (Tamura et al., 2018).

Second, there is variance in performance between images that is a hallmark of the specific computations in the system. For example, one classifier might be exquisitely sensitive to one kind of subtle image difference, but relatively insensitive to the other, whereas a different model, based on different image measurements, would respond differently to the same images. It is this latter source of variance that is of particular interest for computational biology because it reveals the fundamental working of the visual system, rather than a basic characteristic of the input. Future studies should seek to normalize the first source of variance and focus on the second. The purpose of the diagnostic image set was to facilitate this work.

By comparing human performance on the diagnostic image set against an array of models, we were able to show that neither simple color features, nor more sophisticated texture features can predict human judgments of mirror versus glass. More important, we also showed that an arbitrarily defined CNN, which seemed to resemble humans when tested on arbitrary images, did not resemble humans very closely at all when evaluated on the diagnostic image set. The use of arbitrary design decisions is widespread in the literature comparing neural networks with brain activity or human behavior. Our findings suggest that more care should be taking in explicitly searching for, or fitting, neural network models to biological data.

To do this, we then performed a large-scale, systematic search through the space of feedforward networks trained to distinguish mirror from glass objects, in search of a neural network that more closely resembled human performance. The network architecture that performed most similarly to humans (OptCNN) was a three-layer network, which for arbitrary images was not especially good at distinguishing mirror from glass in terms of the true physical labels (at least compared to rival models).

The existence of a set of images that yields systematic errors in humans yet which can be identified correctly by a machine suggests that humans are not optimal at the task in general. If humans were optimal, then images in the diagnostic set that humans misperceive would also lead to errors in any other ideal observer model. Yet, many artificial neural networks can outperform humans, including the default three-layer CNN. It is perhaps not entirely surprising that the human visual system is not optimized for distinguishing mirror from glass, given their infrequency in the natural environment. However, despite many attempts to understand human perceptual processes through normative ideal observer models (Burge, 2020; Geisler, 2011), we suggest that in general, many aspects of material appearance, and perhaps perception more generally, might be better understood as fulfilling objective functions other than optimal estimation of specific distal physical properties (Fleming & Storrs, 2019; Storrs et al., 2021; Storrs & Fleming, 2021).

Despite its poor objective performance at the task of discriminating mirror from glass, OptCNN is the first image-computable model of human perception in this task. Nevertheless, even the optimized model did not reproduce average human behavior on the diagnostic image set as well as individual humans do. Although previous studies have claimed that neural networks reach the noise ceiling of human-to-human performance (Kheradpisheh et al., 2016b; Kubilius et al., 2016), we find that, when tested on a test set that is truly diagnostic of human vision, such conclusions might not in fact be warranted and that further research is required to find good models of human vision.

Why did the OptCNN fail to match human performance? There are at least three important respects in which the models differ from humans and the human brain. First, one of the most striking aspects of human visual cortical processing is the massive amount of feedback (Budd, 1998; Felleman & van Essen, 1991; Mückli & Petro, 2013). It is widely believed that feedforward processing is responsible for many of our visual abilities. For example, high-level visual tasks, such as animal detection (Thorpe, Fize, & Marlot, 1996), can be completed successfully too rapidly for feedback to contribute substantially. Nevertheless, given its anatomical extent, feedback presumably plays an important role in visual processing, potentially in selective visual attention, visual imagery, and the learning process that establishes the representations in the first place. Here, we considered only feedforward architectures. It could be that a key missing ingredient in OptCNN is recurrent processing and that adding feedback signal flow could make up some of the shortfall in correlation with humans. Feedback might, for example, be necessary for performing long-range spatial computations, such as comparing the structures within the region of the object with those of the background, or for example, pooling the disparate local signals into a global sense that glass objects are see-through.

A second important difference is the nature of the training objective. Here—as in almost all neural network-based putative models of human vision (Kriegeskorte, 2015; Majaj & Pelli, 2018; Yamins & DiCarlo, 2016)—we used supervised learning, in which the network is trained on hundreds of thousands of accurately labelled images. Human vision cannot be trained this way, because labelled data are rare (Fleming & Storrs, 2019; Storrs & Fleming, 2021), and the scale of the training set almost certainly exceeds human visual experience with mirror and glass objects. In particular, we very rarely get to see mirror and glass versions of the same objects, and we presumably also exploit the fact that vision unfolds continuously over time, rather than in independent static snapshots, as CNNs are typically trained (Karpathy et al., 2014; van Assen, Nishida, & Fleming, 2020). It is much more likely that visual representations are learned through unsupervised processes, and this may have a critical effect on the internal representations that the visual system learns (Storrs et al., 2021). Neither the networks nor our observers were given feedback on the images they were tested on.

A third important difference between the artificial neural networks and humans lies in the nature of the task that the networks are trained on. Human vision is not tailored solely to the task of distinguishing mirror from glass objects, whereas here, we trained the networks on a binary classification, effectively separating the entire world into two possible states. The representations that optimize performance on this task may well be quite general purpose, as has been found with neural networks optimized for object recognition, which can easily be repurposed for other tasks, such as action recognition (Simonyan & Zisserman, 2014) and image semantic segmentation (Dai, He, & Sun, 2016). Nevertheless, it is also possible that in being trained on such a constrained task, the networks learned representations that do not resemble human visual processes. As a basic test of this, we applied transfer training to the well-known object recognition network AlexNet (Krizhevsky, Sutskever, & Hinton, 2012), replacing the output classification layer with a two-node glass versus mirror classifier. However, the performance was approximately the same as the default three-layer CNN and lower than the OptCNN. Despite this, it would be interesting to test whether a more rigorous approach to obtaining robust, human-like object representations (Geirhos et al., 2018) can lead to human-like performance on material perception tasks.

It is important to emphasize that the similarities to human perception exhibited by OptCNN are restricted to the kinds of images in our training set: computer graphics renditions of a single floating object with uniform glass-like or mirror-like optical characteristics. The networks generalized poorly to images outside this range. For example, we tested the CNN and OptCNN using photographs of real metal and glass objects from the Flickr Material Database (FMD; Sharan, Rosenholtz, & Adelson, 2014) and found that the accuracies of the CNN and OptCNN were 43% and 49%, respectively (in other words, no better than chance). We also observed a strong bias toward interpreting FMD images as glass rather than mirror. One possible reason for the low accuracy is that, unlike our training sets, most of the FMD images are texture-like close-ups and do not have a clear boundary between the object and background. Another possible reason is that the ground truth classes are not identical (mirror and glass in our training set vs. metal and glass in the FMD). Very few of the metal images in the FMD are as specular as the mirror images in our data set. An important direction for future research is to increase generalization by expanding the diversity of the data set.

We suggest that future work should use a combination of unsupervised learning and more naturalistic objective functions and training sets, as well as network architectures that resemble more closely the primate cortex to tease these possibilities apart. We have shown that, for most images, even arbitrarily designed artificial neural networks outperform more conventional hand-engineered models, and thus have substantial potential as models of human visual processes. Nevertheless, when their similarity to humans is investigated with a stricter criterion, they still have important shortcomings. Although neural networks can be found or fitted to the brain or human behavior, out of the box, they should not yet be seen so much as an accurate model of human brain processes, but rather as an experimental platform for further research, much as animal models of neurological disorders are. To make the most of such a platform a crucial additional direction is better tools for probing the inner workings of networks so that further insights can be derived about the specific cues and processes that drive their performance.

## Supplementary Material

Supplement 1
