# Supplementary material for: Distinguishing mirror from glass: A “big data” approach to material perception
Source: J Vis. 2022 Mar 10;22(4):4. doi: 10.1167/jov.22.4.4 (PMC8934559; doi:10.1167/jov.22.4.4)
Supplement: Supplement 1 [file jovi-22-4-4_s001.pdf]

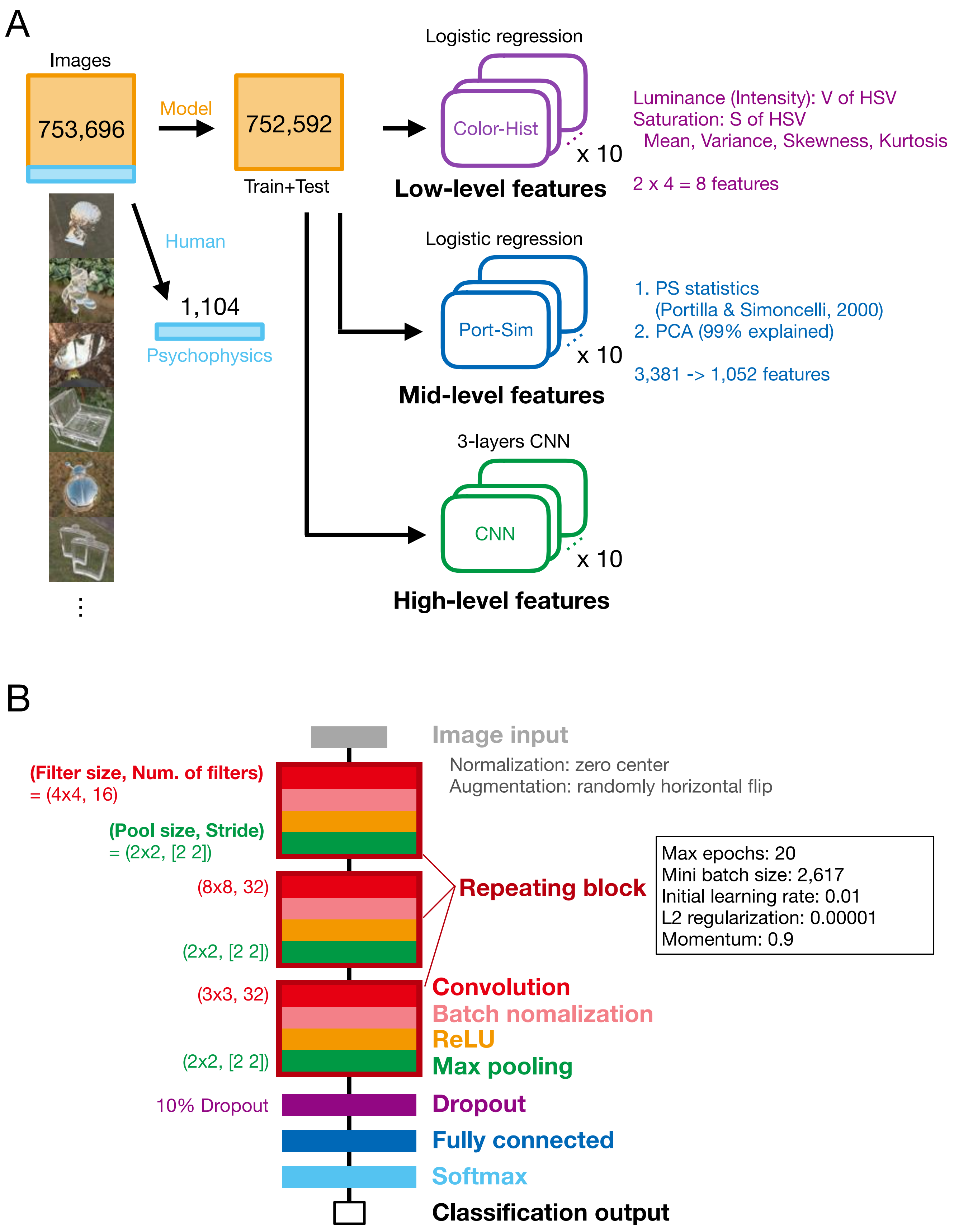

Figure S1. Classifiers and CNN architecture

(A) Flowchart showing development of three classifiers (Color-Hist; Port-Sim and CNN). (B) Network architecture of CNN. The text box shows the hyperparameters for training the CNN. The other hyperparameters were the same as default settings in MATLAB.

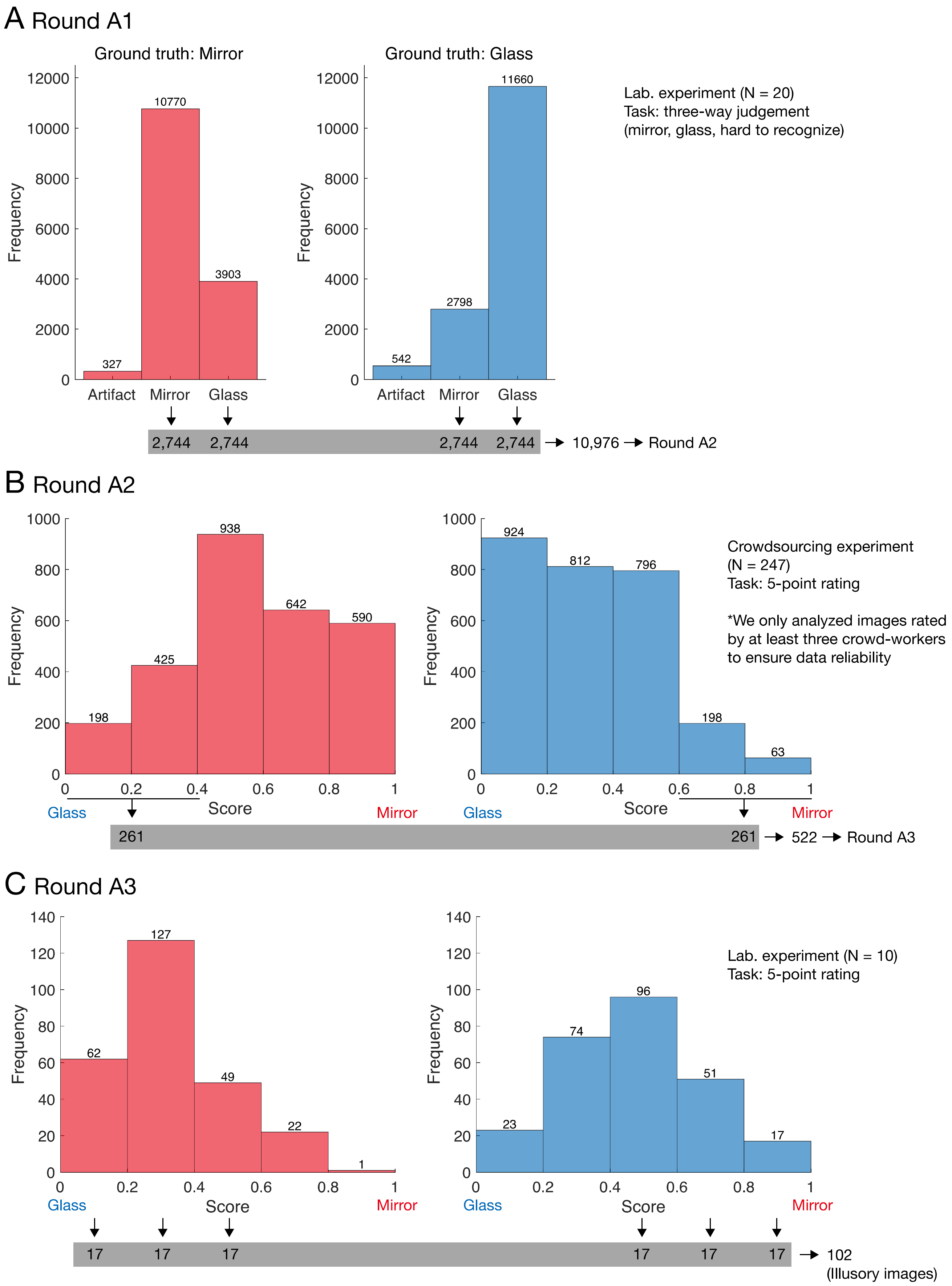

Figure S2. Results of Round A1-A3

Each panel shows the results of each round of experiments as histogram, as in **Figure 2B**. Red bars indicate ground truth mirror renderings; blue indicate ground truth glass renderings. Gray strips indicate number of items from each bin selected for subsequent round. See also **Methods** in Human experiment 2.

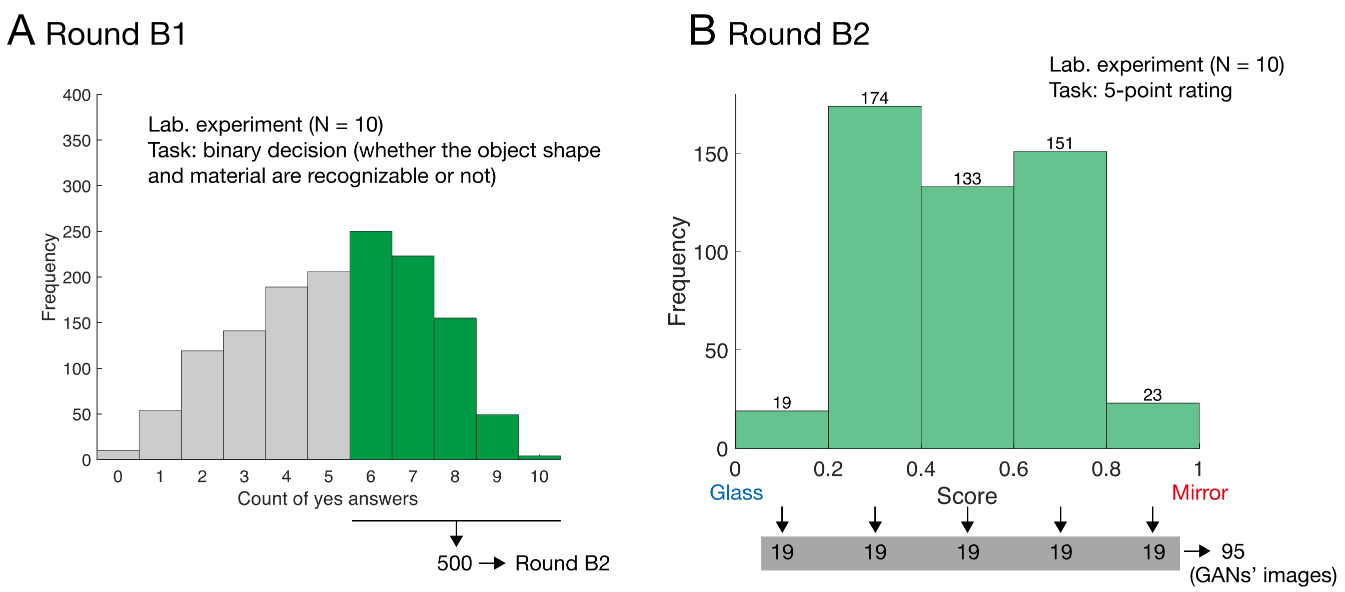

Figure S3. Results of Round B1-B2

Each panel shows result of each round as histogram of images. Green bars indicate GAN images (no ground truth label, unlike in **Figure S2**). Gray strips indicate number of images progressing to subsequent round. See also **Methods** in Human experiment 2.

A

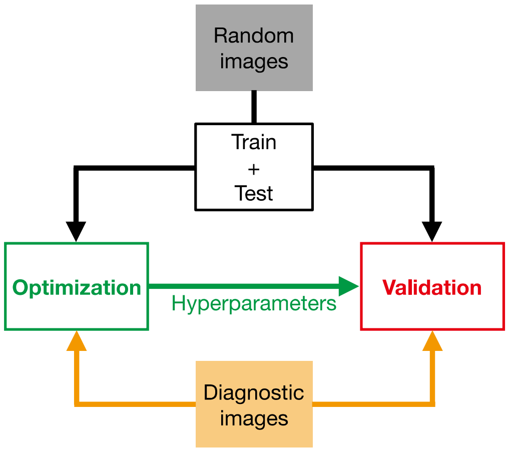

B Optimized hyperparameters

	Name	Min	Max	Type	Transform
1	Initial learning rate	10^{-7}	10^{-1}	Real	Log
2	Factor for L_2 regularization	10^{-7}	10^{-1}	Real	Log
3	Mini batch size	100	2000	Real	-
4	Momentum	0.9	0.96	Real	-
5	Dropout rate	0	0.3	Real	-
6	Filter size 1	2	3	Integer	-
7	Filter size 2	2	4	Integer	-
8	Filter size 3	2	4	Integer	-
9	Num. of filters 1	4	8	Integer	-
10	Num. of filters 2	4	30	Integer	-
11	Num. of filters 3	4	30	Integer	-

Figure S4. Systematic exploration of the space of feedforward networks

(A) Illustration of optimization and validation stages of network exploration. Random renderings are used for training and test during training at both stages. Diagnostic images provide the objective of the Bayesian hyperparameter search, and for testing the trained networks at validation stage. (B) Eleven hyperparameters controlling the network architecture that were adjusted during optimization stage. Each hyperparameter was searched in the range between 'min' and 'max'. The first two hyper-parameters were transformed to log space during the searching. A pair of filter size 1 and num. of filters 1 was set to the convolution layers from the 1st to the n-2 th repeating block. A pair of filter size 2 and num. of filters 2, and a pair of filter size 3 and num. of filters 3 were set to the convolution layer in the penultimate and last repeating blocks. The learning rate was decreased by 0.1 times in each 10 epochs.

Supplement information

Illuminations

https://syns.soton.ac.uk/

http://www.pauldebevec.com/

http://hdrmaps.com/freebies

http://dativ.at/lightprobes/

http://www.openfootage.net/?cat=15

https://hdrihaven.com/hdris.php?thumb=all&sort=date&search=all&page=2&npp=12

https://www.doschdesign.com/
